# A mini-review-cancer energy reprogramming on drug resistance and immune response

**DOI:** 10.1016/j.tranon.2024.102099

**Published:** 2024-08-19

**Authors:** Chengxiang Liu, Liuxin Yang, Tingting Gao, Xingxing Yuan, Ousman Bajinka, Kuanyu Wang

**Affiliations:** a School of Graduate Studies, Heilongjiang University of Chinese Medicine, Harbin 150040, China; b Department of Dermatology, Heilongjiang Academy of Traditional Chinese Medicine, Harbin 150006, China; c Department of Gastroenterology, Heilongjiang Academy of Traditional Chinese Medicine, Harbin 150006, China; d School of Medicine and Allied Health Sciences, University of The Gambia, Banjul, The Gambia; e Department of General Surgery, First Affiliated Hospital of Heilongjiang University of Chinese Medicine, Harbin 150040, China

**Keywords:** Cancer, Energy metabolism, Metabolic reprogramming, Treatment resistance

## Abstract

With the growing interest to harness cancer metabolism and energy reprogramming, this mini review aimed to explain the metabolic programming revealing the mechanisms regarding the treatment resistance. This mini review summarized the prominent cancer metabolic reprogramming on macromolecules. In addition, metabolic reprogramming explaining immune response and treatment resistance as well as energy reprogramming mechanisms are briefly discussed. Finally, some prospects in MR for reversing cancer drug resistance are highlighted.

## Highlights

• This review summarized the prominent cancer metabolic reprogramming on macromolecules.
• In addition, metabolic reprogramming explaining immune response and treatment resistance as well as energy reprogramming mechanisms are briefly discussed.
• Finally, some prospects in MR for reversing cancer drug resistance are highlighted.

## Graphical abstract

**Figure:**
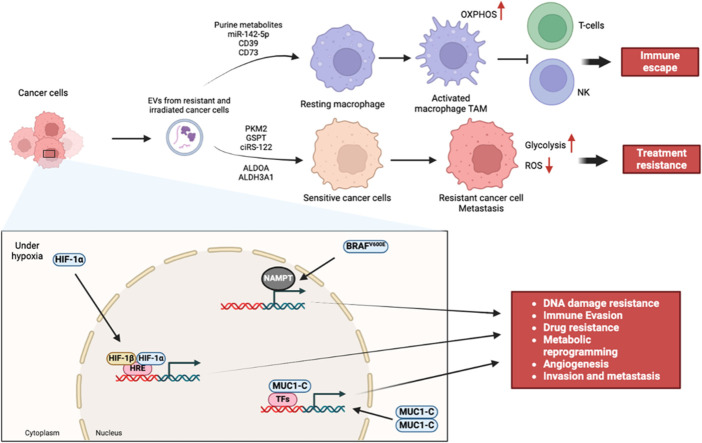

## Introduction

In 2020 alone, almost 10 million cancer related deaths was registered, making it as one of the leading causes of death worldwide. This multifactorial health burden despite the technology, the fight still persists [1]. Cancer energy metabolism that involved mitochondria plays a key role in cancer drug resistance of the hypoxic tumor microenvironment (TME) in cancer cells. With the increasing evidence on immune escape, activation and immune cell metabolism, immunotherapy strategies can be designed on targeting mitochondria [2]. Among the emerging approaches to curb treatment resistance in cancer, targeting metabolism is an effective strategy. Rewiring metabolism in cancer influences tumor-tumor-infiltrating immune cells. Through the modulation of oncogene-targeting, both memory T cells persistence and activation of lymphocytes lead to improved immune-surveillance [3]. With the TME functions, sensitive cells become immune resistance with tumor disease progression [4]. Although there are evidences on metabolic reprogramming (MR) as literature, the recent summation of these findings that could send an informed consent to the researchers regarding the forms of energy reprogramming and the resulting drug resistance is limited. This mini review aimed to shed some lights on the forms of MR inducing the energy metabolism that modulate resistant treatment options for targeted therapy.

## Metabolic reprogramming and treatment resistance

Extracellular vesicles (EVs) as key to drug resistance promote cancer cell proliferation while sustaining angiogenesis, cell invasion, metastasis, MR thus, enabling mutations and modulation of TME [5,6]. In addition, EVs sustain resistance to cancer cell death, reprogram energy metabolism to acquire genome stability [7]. Nearly every characteristic of tumors, including angiogenesis, immune-escape, treatment resistance, and cell proliferation and metastasis, have been linked to tumor-derived EVs. Therapy-resistant cancer cells produce EVs which transfer their genetic material and glycolytic enzymes to sensitive cancer cells. This boosts chemotherapy metabolism, lowers ROS, and improves glycolysis, all of which contribute to treatment resistance. Purine metabolites, genetic material, and glycolytic enzymes that polarize macrophages to tumor-associated macrophages (TAMs) are carried and delivered by EVs, and these factors, directly impair the immune response to cancer [8]. In this light, once EVs signaling networks are hijacked, it will lead to anti-tumor for a variety of cancers through obstructing malignant communications. In addition to glycolysis and oxidative phosphorylation, MR is a key process that facilitates HIF target genes functions [9]. Although HIFs induced resistance has a very limited literature, phytochemicals and chemotherapy are promising for patients with HIF-1-independent drug resistance mechanisms [10]. HIF-1α/estrogen-related receptor α (HIF-1α/ERRα) promotes pyroptosis resistance and enhances tumor growth adaptation [11]. Through aerobic glycolysis as the main pathway, cancer cells energy reprogramming is utilizing anabolic functions for metabolic energy. One specific MR is the upregulation of ATPase inhibitory factor 1 (IF1), the inhibitor of the H^+^-ATP synthase [12]. Limiting ATP production, overexpression of IF1 enhances glycolysis through energy reprogramming metabolism. The mitochondrial reactive oxygen species (mtROS) that modulates the pathway signaling molecule for cellular proliferation and invasion is promoted, thereby modulating tumor immune response and thus resistance to cell death.

A diverse nutritional supplement and reduced pH value is induced by nasopharyngeal carcinoma (NPC) [13]. This is evident with tumor-infiltrating TME that incorporate fibroblast, immune cells and endothelial cells to facilitate immunosuppression and progression of cancer. The associating treatment resistance can be checked through MR and immune system study. TME in some cancers for instance, pancreatic cancer (PC) enables treatment resistance in addition to tumor growth, metastasis and immune response suppression. Cancer spread and disease lapses are strongly associated with PC increased resistance with distinct metabolic properties [14]. Although not conclusive, bladder cancer associated with increased drug resistance is studied to be influenced by type I transmembrane protein, MUC1 through immune cell infiltration and metabolism [15]. Importin-β facilitates the import of MUC1-C homodimers into the nucleus where it interacts with various transcription factors (TFs) including WNT/β-catenin/TCF4, NF-κB, NOTCH, and MYC, to promote epigenetic reprogramming, and epithelial–mesenchymal transition (EMT) process. These processes support immune evasion and DNA damage resistance [16].

Warburg effect as a key cancer progression machinery is an active metabolic reprogramming [17]. Warburg effects defined a unique TME, which is epigenetically cooperated with hypoxia-inducible factor-1 (HIF-1) [18]. Its effect emerged from HIF-1 overexpression (normoxic or hypoxic) as well as oncogene activation especially with cMyc, Ras. Under hypoxic conditions, HIF-1α accumulates and subsequently moves into the nucleus and form a complex with HIF-β that binds to hypoxia response elements (HRE) in the promoter region of genes engaged in angiogenesis, survival and proliferation. In addition, it affirms oncogene activation and loss of function of tumor through adenosine triphosphate (ATP) generation, diversion of glycolytic intermediates, inhibition of pyruvate, accumulation of lactate and glycolytic fluxes acceleration [19]. In addition to the 6 biological process as the hallmark of cancer such as cell proliferation, evasion of growth suppressors, resistance, immortality, angiogenesis, invasion and metastasis, the RE-1 silencing transcription factor or neuron restrictive silencing factor NRSF/REST are essential targets for cancer therapy [20].

Under covering the carbon and glucose utilization as an alternative source for cancer growth explained mitochondrial oxidative phosphorylation (OXPHOS) metabolism [21]. OXPHOS revealed the interplay between immune cells, neoplastic cells and stroma to participate in resistance to treatments through tumorigenesis [22]. Another metabolic reprogramming associated with the immune cell microenvironment are carbohydrate and lipid pathways, forming a crucial prognosis for gynecological cancer [23]. MR and therapeutic resistance depend on multiple processes, notably cancer metabolism, the expression of cancer genes, their epigenetic modifications and the energy requests of TME. Although there has been evidence to support the Warburg effect, the cancer drug resistance detecting biomarkers are still missing.

**Fig. 1:**
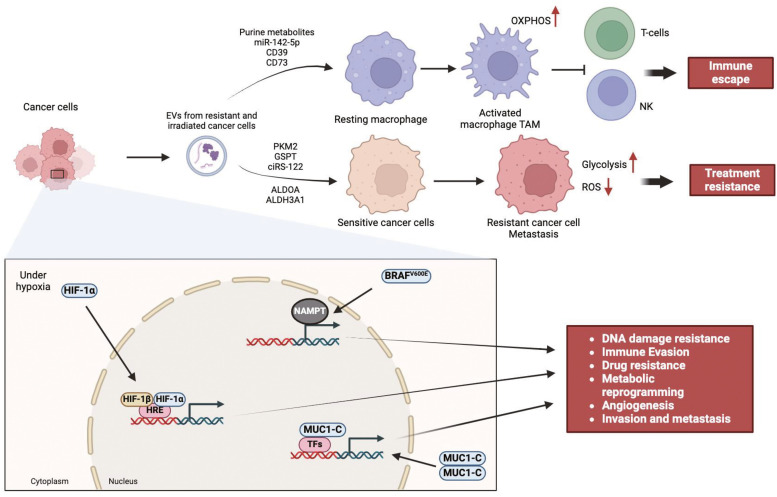
Metabolic reprogramming treatment resistance and immune escape processes in cancer cells.

For melanoma progression, nicotinamide phosphoribosyltransferase (NAMPT) as an energy limiting enzyme of nicotinamide adenine dinucleotide (NAD) metabolism is now studied for its role in targeted therapy resistance [24]. NAMPT transcription and NAD metabolism are enhanced by the activation of the BRAF-mutated pathway. Elevated NAMPT and NAD concentrations support an energy metabolism and fuel drug resistance processes. In addition, rapamycin (mTOR) pathway as a mammalian target can regulate functions of melanoma cell anabolism and energy metabolism and these are evident for both resistance and sensitive therapy. Treatment resistance in cancer is induced by immunosuppression and antitumor immunity impairment. This is significantly prominent with tumor progression through MR dynamics with distinct immune responses leading to a new emerging discipline called immunometabolism. In addition to metabolic display, nutrient competition existing between infiltrating immune cells and tumor cells confers explanations to antitumor immunity [25] (Fig. 1 and Table 1).

**Table 1:** Cancer energy reprogramming on drug resistance and immune response.

Objective	Method	Main finding	Conclusions	Refs.
Role of ATP in cancer immunosurveillance	▪ Nanocarriers for tumor targeting delivery	▪ Increases in pro-inflammatory eATP. ▪ Blocking immunosuppressive adenosine and alleviating hypoxia.	▪ An innovative strategy to advance eATP-driven antitumor immunity.	[26]
Mechanism of MUC1 in bladder cancer	▪ TCGA-BLCA ▪ (GES31684 and GSE13507)	▪ The gene is associated with immune response, ribosomes, and exosomes. ▪ Through MR expression of MUC1 increases, drug resistance, glycolysis, glucose uptake and lactate production.	▪ The increased drug resistance result to this process.	[27]
Toxicity of free small molecule inhibitor combinations	▪ TME activatable prodrug nanomicelle (PNM)	▪ Gasdermin E (GSDME)-based immunogenic pyroptosis of tumor cells was triggered to elicit a robust antitumor immune response.	▪ Pyroptosis-induced nanoplatform codelivery of PI3K/mTOR and CDK inhibitors reprogram the immunosuppressive TME and efficiently improve checkpoint blockade cancer immunotherapy.	[28]
Preclinical characterization of Halofuginone	▪ LC-MS/MS analysis	▪ Halofuginone markedly reduced the viability of cancer cells with systemic toxicity relieved. ▪ Free Halofuginone-suppressive properties is maintained.	▪ A magnitude of adverse effects has reduced. ▪ Halofuginone micelle can potentially counteract Nrf2-activated cancers.	[29]
The metabolic subtypes of cervical carcinoma	▪ TCGA and GEO datasets	▪ Immune infiltration is strongly linked to the metabolic pathways. ▪ These are evident with carbohydrate and lipid energy pathway.	▪ Immune cell microenvironment is a potential therapeutic target.	[30]
Prostate cancer patients on androgen deprivation therapy	▪ RCT	▪ Aerobic capacity decreased.	▪ Resistance exercise training counteracts the adverse effects of androgen deprivation therapy.	[31]
Ketogenic diet and endometrial cancer	▪ RCT	▪ An inverse association between the changes in serum β-hydroxybutyrate and IGF-I concentrations.	▪ Serum β-hydroxybutyrate may reflect MR of cancer.	[32]
Biological mechanisms of chemoradiotherapy for head and neck cancer	▪ RCT	▪ Improved quality of life and reduced adverse effects.	▪ Concomitant chemoradiotherapy may curb resistance.	[33]
Resistance exercise in gastrointestinal cancer on chemotherapy	▪ RCT	▪ Fatigue and appetite loss were significantly lowered.	▪ Resistance exercise training alleviated fatigue and appetite loss in gastrointestinal cancer.	[34]
Combined approach of training and protein supplementation Prostate cancer patients on androgen deprivation therapy	▪ Pilot RCT	▪ No significant differences.	▪ No changes in MetS or physical function.	[35]
Acute changes for resistance exercise in chemotherapy for breast cancer	▪ Randomized crossover equivalence trial	▪ Findings from this study are relevant for exercise prescription guidelines.	▪ Low- and heavy-load resistance exercise elicited similar acute responses in arm swelling and breast cancer-related lymphedema symptoms in women at risk for lymphedema receiving adjuvant taxane-based chemotherapy.	[36]
Resistance exercise program in women treated for premenopausal breast cancer	▪ RCT	▪ Bone turnover markers decreased.	▪ No prevent bone loss among women.	[37]
Chronic lymphocytic leukemia and treatment resistance	▪ Experimental (*in vivo*)	▪ Transcriptional downregulation of pathways mediating energy metabolism.	▪ Early treatment period is important drug-resistant.	[38]

## Metabolic reprogramming immune response

In cancer research immunometabolism can shift the metabolic competition to the favor of immune cells, reverse treatment resistance and initiate anti-tumor mechanisms during the treatment [39]. MR resulting from TME affects both the immune cells and their immunosurveillance, thereby checking the cancer treatment resistance [40]. TME acidity regulates tumor immunity through influencing immune cells metabolism, thus contributing to the systemic immunity and enhancing tumor progression and subsequently therapeutic resistance. Among the immune cells include myeloid-derived suppressor cells (MDSC) are TAMs and dendritic cells [41]. Moreover, antitumor immune responses face resistance due to oncogenic signaling [42].

Hypoxia from excessive tumor growth regulate TME in the favor of cancer such as enhanced glycolysis in producing metabolites for cancer cells [43]. Both fatty acids and glutamine uptake are increased due to hypoxic cancer cells metabolism as in hexosamine biosynthesis pathway (HBP). The resulting signaling pathway called protein post-translational modification (PTM) releases UDP-GlcNAc as the end product to facilitate tumor progression [44]. Such metabolic changes in TME affect tumor immune cells through tumor immunosuppressive microenvironment thereby inducing resistance to immunotherapy [45].

## Cancer metabolic reprogramming on macromolecules

As a precursor of gluconeogenesis, the glycolysis end product lactate is also a signaling molecule. Both cancer progression of cancer and the associated drug resistance are epigenetically linked to lactate reprogrammed energy input. These changes are ascribed in histone lysine lactylation and lactate-induced histone modification [46]. Therapeutic resistance through altered amino acid metabolism leads to tumor outgrowth as described in immune response. Immune cell differentiations are triggered through amino acid metabolic enzymes such as GCN2 and mTOR. Therefore, it will be a metabolic advantage for cells against cancer cells through amino acid energy rewiring through targeting enzymes and their sensors [47].

As a selfish metabolic mechanism, cancer cells switch to abnormal metabolism thereby initiating energy tradeoffs between its essential function and resistance. One specific with growing attention is the lipid where choline metabolism has enough mechanism for resistance through the phosphatidylcholine cycle. In the prospective cancer research, phosphatidylcholine metabolism as in response to stress will shed some light to cancer therapy resistance [48]. Phosphatidylcholine source of energy also forms an integral part of cell membrane constituents. This lipid plays a key role in cellular communication between immune cells and cancer cells in TME. To this end, an immune regulatory mechanism is conferred by this lipid in therapeutic functions [49].

## Prospects in metabolic reprogramming for reversing drug resistance

The increased production of lactate is also prominent for nitric oxide, prostaglandins and ROS as well as arachidonic acid metabolism. These energy metabolism influence both TAMs and TME thus tumor cell metabolism. TAM will eventually lead to production of angiogenic factors and cytokines that contribute to the progression of tumor growth and metastasis. Understanding TAMs recruitment of these processes can help to potentiate anti-tumor and reversing treatment resistance therapy [50]. A master regulator for cellular response against xenobiotic and oxidative stress, the system of Keap1-Nrf2 induces metabolic reprogramming thereby making a highly regarded anti-resistance therapeutic strategy [51].

There is an innovative approach that revealed advanced strategies on pro-inflammatory extracellular ATP (eATP) [26]. Increased eATP potentiate antigen capacity of DCs to enable NK cells and T cells functions. This is done through driving macrophages pyroptosis from P2 × 7-NLRP3-inflammasome activation. Subsequently, tumor progression is suppressed through the synergistic antitumor immune response thereby inhibiting distant metastases and reverse anti-PD1 resistance. The most promising for cancer biochemistry in quest for rapid and non-recurrent, non-treatment resistance cancers is the mechanistic study of the association between tumor immunity and MR and the therapeutic strategy [52]. For instance, there should be guidelines to interpret autophagy and related processes. Autophagy based therapy has growing prospects in overcoming tumor resistance to treatment strategies [53]. T cell energy reprogramming can reverse T cell dysfunction and check treatment resistance to the number of cancer types [54]. The directives involving cancer stem cells to dictate TME in their favors via growth factors releases, chemokines and cytokines promote neo-angiogenesis could be explored for harnessing treatment failures due to tumor drug resistance [55].

**Fig. 2:**
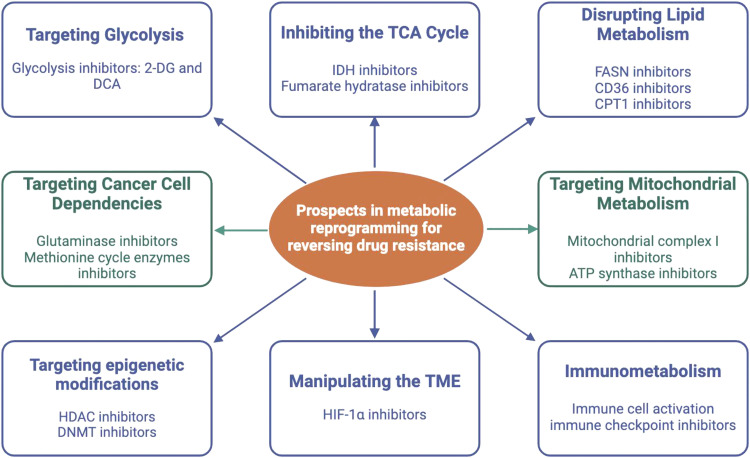
Various prospects in metabolic reprogramming for reversing treatment resistance in cancers.

The ability of cancer cells to modify their metabolism in order to sustain fast growth and survival is known as MR, and it frequently leads to treatment resistance. Targeting glycolysis, TCA cycle, lipid metabolism, mitochondria metabolism, cancer cell dependencies, metabolic epigenetic regulations, nutrient availability in the TME and immunometabolism are some significant prospects and approaches for drug resistance reversal via MR. Even in the presence of oxygen, many cancer cells use glycolysis as a source of energy (the Warburg effect), thus, interfering with this metabolic route by using glycolysis inhibitors like dichloroacetate (DCA) and 2-deoxyglucose (2-DG) may be able to reverse resistance. Isocitrate dehydrogenase (IDH) and fumarate hydratase are two important TCA cycle enzymes that can be targeted to disrupt metabolic pathways that are essential for resistant cancer cells. Fatty acid production is frequently upregulated in cancer cells. Fatty acid synthase (FASN) inhibitors have the ability to decrease lipid availability, which might hinder energy storage and the formation of cell membranes. By targeting pathways related to oxidation (CPT1 inhibitors) and lipid uptake (CD36 inhibitors), cancer cells' metabolic flexibility can be decreased, increasing their susceptibility to chemotherapy treatments. It is common to find that cancer cells are dependent on specific amino acids, like glutamine. Cancer cells can be starved of glutamine, which is necessary for the production of nucleotides and proteins, using glutaminase inhibitors (CB-839). Drug-resistant metabolic adaptations can be reversed by focusing on epigenetic changes that control metabolic gene expression. For example, drugs that block DNA methyltransferases (DNMTs) or histone deacetylases (HDACs) may restore chemotherapy efficacy. Improving immune cells' (like T cells') metabolic fitness with metabolic modulators can strengthen their capacity to combat cancer cells. In addition, anti-tumor immune responses can be boosted by combining metabolic therapies with immune checkpoint inhibitors such as anti-PD-1/PD-L1 (Fig. 2).

## Conclusion

It is apparent that strong evidence is now emerging to confer progress on the recent cancer treatment strategies and prevention based on MR. This change in energy acquisition is studied with promising explanations to distinct mechanisms of cancers, especially the types that pose treatment resistance. To this end, targeted therapies to reverse drug resistance mechanisms should be based on the metabolism influenced by cancer TME.

## Ethics approval and consent to participate

Not applicable.

## Funding

Excellent Youth Project of Natural Science Foundation of Heilongjiang Province (No: YQ2022H015); Research project of Heilongjiang Provincial Health Commission (No: 20220404010995).

## CRediT authorship contribution statement

**Chengxiang Liu:** Conceptualization. **Liuxin Yang:** Data curation. **Tingting Gao:** Writing – review & editing. **Xingxing Yuan:** Writing – original draft. **Ousman Bajinka:** Writing – review & editing. **Kuanyu Wang:** Writing – original draft, Conceptualization.

## Declaration of competing interest

The authors declare that they have no known competing financial interests or personal relationships that could have appeared to influence the work reported in this paper.

## References

[1] Jahan S., Mukherjee S., Ali S., Bhardwaj U., Choudhary R.K., Balakrishnan S., Naseem A., Mir S.A., Banawas S., Alaidarous M. (2022). Pioneer role of extracellular vesicles as modulators of cancer initiation in progression, drug therapy, and vaccine prospects. Cells.

[2] Bai R., Cui J. (2023). Mitochondrial immune regulation and anti-tumor immunotherapy strategies targeting mitochondria. Cancer Lett..

[3] Fumarola C., Petronini P.G., Alfieri R. (2018). Impairing energy metabolism in solid tumors through agents targeting oncogenic signaling pathways. Biochem. Pharmacol..

[4] El Sayed R., Haibe Y., Amhaz G., Bouferraa Y., Shamseddine A. (2021). Metabolic factors affecting tumor immunogenicity: what is happening at the cellular level?. Int. J. Mol. Sci..

[5] Xavier C.P.R., Caires H.R., Barbosa M.A.G., Bergantim R., Guimaraes J.E., Vasconcelos M.H. (2020). The role of extracellular vesicles in the hallmarks of cancer and drug resistance. Cells.

[6] Fridman E.S., Ginini L., Gil Z. (2022). The role of extracellular vesicles in metabolic reprogramming of the tumor microenvironment. Cells.

[7] Yang E., Wang X., Gong Z., Yu M., Wu H., Zhang D. (2020). Exosome-mediated metabolic reprogramming: the emerging role in tumor microenvironment remodeling and its influence on cancer progression. Signal. Transduct. Target. Ther..

[8] Gunassekaran G.R., Baek M.C., Lee B., Poongkavithai Vadevoo SM (2021). M1 macrophage exosomes engineered to foster M1 polarization and target the IL-4 receptor inhibit tumor growth by reprogramming tumor-associated macrophages into M1-like macrophages. Biomaterials.

[9] Kierans S.J., Taylor C.T. (2021). Regulation of glycolysis by the hypoxia-inducible factor (HIF): implications for cellular physiology. J. Physiol..

[10] Ma Z., Xiang X., Li S., Xie P., Gong Q., Goh B.C., Wang L. (2022). Targeting hypoxia-inducible factor-1, for cancer treatment: Recent advances in developing small-molecule inhibitors from natural compounds. Semin. Cancer Biol..

[11] Su P., Yu L., Mao X., Sun P. (2022). Role of HIF-1alpha/ERRalpha in enhancing cancer cell metabolism and promoting resistance of endometrial cancer cells to pyroptosis. Front. Oncol..

[12] Esparza-Molto P.B., Cuezva J.M. (2018). The role of mitochondrial H(+)-ATP synthase in cancer. Front. Oncol..

[13] Huang H., Li S., Tang Q., Zhu G. (2021). Metabolic reprogramming and immune evasion in nasopharyngeal carcinoma. Front. Immunol..

[14] Zuzcak M., Trnka J. (2022). Cellular metabolism in pancreatic cancer as a tool for prognosis and treatment (Review). Int. J. Oncol..

[15] Hosseinzadeh A., Merikhian P., Naseri N., Eisavand M.R., Farahmand L. (2022). MUC1 is a potential target to overcome trastuzumab resistance in breast cancer therapy. Cancer Cell Int..

[16] Yamashita N., Kufe D. (2022). Addiction of cancer stem cells to MUC1-C in triple-negative breast cancer progression. Int. J. Mol. Sci..

[17] Vaupel P., Schmidberger H., Mayer A. (2019). The Warburg effect: essential part of metabolic reprogramming and central contributor to cancer progression. Int. J. Radiat. Biol..

[18] Vaupel P., Multhoff G. (2021). The warburg effect: historical dogma versus current rationale. Adv. Exp. Med. Biol..

[19] Vaupel P., Multhoff G. (2021). Revisiting the Warburg effect: historical dogma versus current understanding. J. Physiol..

[20] Arizmendi-Izazaga A., Martinez-Baltazar R., Liborio-Bautista A., Olea-Flores M., Ortiz-Ortiz J., Navarro-Tito N. (2023). The NRSF/REST transcription factor in hallmarks of cancer: From molecular mechanisms to clinical relevance. Biochimie.

[21] Ashton T.M., Mckenna W.G., Kunz-Schughart L.A., Higgins G.S. (2018). Oxidative phosphorylation as an emerging target in cancer therapy. Clin. Cancer Res..

[22] Luo Y., Ma J., Lu W. (2020). The significance of mitochondrial dysfunction in cancer. Int. J. Mol. Sci..

[23] Hr J., Wang J., Wang Z.J., Xi M.J., Xia B.H., Deng K., Yang J.L. (2023). Lipid metabolic reprogramming in tumor microenvironment: from mechanisms to therapeutics. J. Hematol. Oncol..

[24] Indini A., Fiorilla I., Ponzone L., Calautti E., Audrito V. (2022). NAD/NAMPT and mTOR pathways in melanoma: drivers of drug resistance and prospective therapeutic targets. Int. J. Mol. Sci..

[25] Li X., Xu W. (2019). CD147‑mediated reprogrammed glycolytic metabolism potentially induces immune escape in the tumor microenvironment (Review). Oncol. Rep..

[26] Wu L., Xie W., Li Y., Ni Q., Timashev P., Lyu M., Xia L., Zhang Y., Liu L., Yuan Y. (2022). Biomimetic nanocarriers guide extracellular ATP homeostasis to remodel energy metabolism for activating innate and adaptive immunity system. Adv. Sci..

[27] Qing L., Li Q., Yang Y., Xu W., Dong Z. (2022). A prognosis marker MUC1 correlates with metabolism and drug resistance in bladder cancer: a bioinformatics research. BMC Urol..

[28] Yang Q., Ma X., Xiao Y., Zhang T., Yang L., Yang S., Liang M., Wang S., Wu Z., Xu Z. (2022). Engineering prodrug nanomicelles as pyroptosis inducer for codelivery of PI3K/mTOR and CDK inhibitors to enhance antitumor immunity. Acta Pharm. Sin. B.

[29] Panda H., Suzuki M., Naito M., Saito R., Wen H., Baird L., Uruno A., Miyata K., Yamamoto M. (2022). Halofuginone micelle nanoparticles eradicate Nrf2-activated lung adenocarcinoma without systemic toxicity. Free Radic. Biol. Med..

[30] Dai G., Ou J., Wu B. (2022). A predictive study of metabolism reprogramming in cervical carcinoma. Ann. Transl. Med..

[31] Houben L.H.P., Overkamp M., Vank P., Trommelen J., Vanr J.G.H., Dev P., Del K., Vdm S., Mikkelsen U.R., Verdijk L.B. (2023). Resistance exercise training increases muscle mass and strength in prostate cancer patients on androgen deprivation therapy. Med. Sci. Sports Exerc..

[32] Cohen C.W., Fontaine K.R., Arend R.C., Alvarez R.D., Leath C.A., Huh W.K., Bevis K.S., Kim K.H., Straughn J.M., Gower B.A. (2018). A ketogenic diet reduces central obesity and serum insulin in women with ovarian or endometrial cancer. J. Nutr..

[33] Lonkvist C.K., Lonbro S., Vinther A., Zerahn B., Rosenbom E., Primdahl H., Hojman P., Gehl J. (2017). Progressive resistance training in head and neck cancer patients during concomitant chemoradiotherapy – design of the DAHANCA 31 randomized trial. BMC Cancer.

[34] Hong Y., Wu C., Wu B. (2020). Effects of resistance exercise on symptoms, physical function, and quality of life in gastrointestinal cancer patients undergoing chemotherapy. Integr. Cancer Ther..

[35] Dawson J.K., Dorff T.B., Todd Schroeder E., Lane C.J., Gross M.E. (2018). Dieli-Conwright CM: impact of resistance training on body composition and metabolic syndrome variables during androgen deprivation therapy for prostate cancer: a pilot randomized controlled trial. BMC. Cancer.

[36] Bloomquist K., Oturai P., Steele M.L., Adamsen L., Moller T., Christensen K.B., Ejlertsen B., Hayes S.C. (2018). Heavy-load lifting: acute response in breast cancer survivors at risk for lymphedema. Med. Sci. Sports Exerc..

[37] Tabatabai L.S., Bloom J., Stewart S., Sellmeyer D.E. (2019). A randomized controlled trial of exercise to prevent bone loss in premenopausal women with breast cancer. J. Womens Health.

[38] Landau D.A., Sun C., Rosebrock D., Herman S.E.M., Fein J., Sivina M., Underbayev C., Liu D., Hoellenriegel J., Ravichandran S. (2017). The evolutionary landscape of chronic lymphocytic leukemia treated with ibrutinib targeted therapy. Nat. Commun..

[39] Sung J.Y., Cheong J.H. (2022). New immunometabolic strategy based on cell type-specific metabolic reprogramming in the tumor immune microenvironment. Cells.

[40] Kanwore K., Kanwore K., Adzika G.K., Abiola A.A., Guo X., Kambey P.A., Xia Y., Gao D. (2022). Cancer metabolism: the role of immune cells epigenetic alteration in tumorigenesis, progression, and metastasis of glioma. Front. Immunol..

[41] Hasan M.N., Capuk O., Patel S.M., Sun D. (2022). The role of metabolic plasticity of tumor-associated macrophages in shaping the tumor microenvironment immunity. Cancers.

[42] Kobayashi Y., Lim S.O., Yamaguchi H. (2020). Oncogenic signaling pathways associated with immune evasion and resistance to immune checkpoint inhibitors in cancer. Semin. Cancer Biol..

[43] Li Y., Zhao L., Li X.F. (2021). Hypoxia and the tumor microenvironment. Technol. Cancer Res. Treat..

[44] Paneque A., Fortus H., Zheng J., Werlen G., Jacinto E. (2023). The hexosamine biosynthesis pathway: regulation and function. Genes.

[45] Hao X., Ren Y., Feng M., Wang Q., Wang Y. (2021). Metabolic reprogramming due to hypoxia in pancreatic cancer: implications for tumor formation, immunity, and more. Biomed. PharmacOther.

[46] Chen A.N., Luo Y., Yang Y.H., Fu J.T., Geng X.M., Shi J.P., Yang J. (2021). Lactylation, a novel metabolic reprogramming code: current status and prospects. Front. Immunol..

[47] Yang L., Chu Z., Liu M., Zou Q., Li J., Liu Q., Wang Y., Wang T., Xiang J., Wang B. (2023). Amino acid metabolism in immune cells: essential regulators of the effector functions, and promising opportunities to enhance cancer immunotherapy. J. Hematol. Oncol..

[48] Saito R.F., Andrade L.N.S., Bustos S.O., Chammas R. (2022). Phosphatidylcholine-derived lipid mediators: the crosstalk between cancer cells and immune cells. Front. Immunol..

[49] Ma Y., Zhang S., Jin Z., Shi M. (2020). Lipid-mediated regulation of the cancer-immune crosstalk. Pharmacol. Res..

[50] Zheng J., Jiang J., Pu Y., Xu T., Sun J., Zhang Q., He L., Liang X. (2023). Tumor-associated macrophages in nanomaterial-based anti-tumor therapy: as target spots or delivery platforms. Front. Bioeng. Biotechnol..

[51] Taguchi K., Yamamoto M. (2020). The KEAP1-NRF2 system as a molecular target of cancer treatment. Cancers. (Basel).

[52] Klionsky D.J., Abdel-Aziz A.K., Abdelfatah S., Abdellatif M., Abdoli A., Abel S., Abeliovich H., Abildgaard M.H., Abudu Y.P., Acevedo-Arozena A. (2021). Guidelines for the use and interpretation of assays for monitoring autophagy. Autophagy..

[53] Tang L., Zhang H., Zhou F., Wei Q., Du M., Wu J., Li C., Luo W., Zhou J., Wang X. (2024). Targeting autophagy overcomes cancer-intrinsic resistance to CAR-T immunotherapy in B-cell malignancies. Cancer Commun..

[54] Xuekai L., Yan S., Jian C., Yifei S., Xinyue W., Wenyuan Z., Shuwen H., Xi Y. (2024). Advances in reprogramming of energy metabolism in tumor T cells. Front. Immunol..

[55] Naik P.P., Panda P.K., Bhutia S.K. (2017). Oral cancer stem cells microenvironment. Adv. Exp. Med. Biol..

